# Acceleration of Functional Maturation and Differentiation of Neonatal Porcine Islet Cell Monolayers Shortly In Vitro Cocultured with Microencapsulated Sertoli Cells

**DOI:** 10.4061/2010/587213

**Published:** 2009-11-19

**Authors:** Francesca Mancuso, Mario Calvitti, Giovanni Luca, Claudio Nastruzzi, Tiziano Baroni, Stefania Mazzitelli, Ennio Becchetti, Iva Arato, Carlo Boselli, Monique D. Ngo Nselel, Riccardo Calafiore

**Affiliations:** ^1^Section of Internal Medicine and Endocrine and Metabolic Sciences, Department of Internal Medicine, University of Perugia, 06126 Perugia, Italy; ^2^Department of Experimental Medicine and Biochemical Sciences, University of Perugia, 06126 Perugia, Italy; ^3^Department of Chemistry and Technology of the Drug, School of Pharmacy, University of Perugia, 06126 Perugia, Italy; ^4^Department of Surgery, University of Perugia, 06126 Perugia, Italy

## Abstract

The limited availability of cadaveric human donor pancreata as well as the incomplete success of the Edmonton protocol for human islet allografts fasten search for new sources of insulin the producing cells for substitution cell therapy of insulin-dependent diabetes mellitus (T1DM). Starting from isolated neonatal porcine pancreatic islets (NPIs), we have obtained cell monolayers that were exposed to microencapsulated monolayered Sertoli cells (ESCs) for different time periods (7, 14, 21 days). To assess the development of the cocultured cell monolayers, we have studied either endocrine cell phenotype differentiation markers or c-kit, a hematopoietic stem cell marker, has recently been involved with growth and differentiation of *β*-cell subpopulations in human as well as rodent animal models. ESC which were found to either accelerate maturation and differentiation of the NPIs *β*-cell phenotype or identify an islet cell subpopulation that was marked positively for c-kit. The insulin/c-kit positive cells might represent a new, still unknown functionally immature *β*-cell like element in the porcine pancreas. Acceleration of maturation and differentiation of our NPI cell monolayers might generate a potential new opportunity to develop insulin-producing cells that may suite experimental trials for cell therapy of T1DM.

## 1. Introduction

Correction of hyperglycemia by exogenous insulin may delay or attenuate, but never eliminate, the risk for developing secondary complications during the time course of T1DM [1]. Moreover, recently possible risks for malignancies in diabetic patients treated with long-lasting insulin analogues have been reported [2]. Transplantation (TX) of insulin-producing tissue, whether being comprised of whole pancreas or isolated islet cells, might fully restore normoglycemia, thus obviating the need for daily exogenous insulin supplementation. Moreover, the improved stability of metabolic control, as achieved by islet TX, could restrain both the onset and severity of the disease-related chronic complications. Nevertheless, human islet allografts in totally immunosuppressed patients with T1DM have been proven quite limited, in terms of successful outcome [3]. The majority of long-term transplanted islets failed to become fully functional and sustain the transiently restored euglycemia [3]. In addition, availability of human donor islets usually is very restricted due to low organ donation rate which does warrant looking for alternative cell sources like neonatal porcine islets (NPIs), as human tissue substitutes [4–7].

In fact, meanwhile it is very difficult to obtain and culture maintain adult porcine islets because of their intrinsic fragility and short functional life-span, in contrast, NPIs procurement is very easy, and above all, NPIs grow and differentiate easier than adult porcine islets, have a long functional life-span, and are protected from CD8+ attack [8]. 

Currently, a major hurdle to use NPI for xenograft is the delay in achieving the posttransplant normalization of blood glucose in diabetic recipients. In fact, as reported by Korbutt et al. [9] and Korbutt et al. [4], NPI may take 4–10 or more weeks to reach as sufficient, differentiated *β*-cell mass as to enable reversal of hyperglycemia after TX in diabetic rodents. 

This pre-TX time-lag poses two major problems: (a) recipients need to be treated with exogenous insulin, until the graft becomes functional; (b) the implanted islets are exposed to environmental, as much as detrimental, chronic hyperglycemia. 

Several efforts have been made to accelerate the lengthy NPI maturation process, both in vivo and in vitro, but these procedures unfortunately are associated with relevant cell mass loss.

Freshly isolated NPI cell populations are typically comprised of a minority of *β*-cells, and a majority of cytokeratin-7+ (Ck7, a ductal cell marker) cells. The remaining cells coexpress either insulin, or epithelial cell markers [5] or pancreatic and duodenal homeobox gene-1 (PDX-1), which regulates glucose-stimulated insulin gene expression [9]. In this respect, we had previously shown that coincubation of NPI with Sertoli cells (SCs) induced rapid and significant maturation and differentiation of NPI immature into functionally competent, mature *β*-cells, by promoting acceleration of the differentiation/developmental process [5].

The assumption was that SC would provide nutrients and immunomodulatory and trophic factors that were likely to improve survival and development as well as functional competence of NPI [5, 10]. To ameliorate SC effects on NPI, we have developed a culture procedure to obtain NPI monolayers (maintained in vitro up to 90 days) treated with high level glucose and glucagon-like peptide 1 (GLP-1) [11]. 

In order to ascertain mechanisms underlying SC-driven acceleration of the NPI cell monolayer differentiation, we incubated NPI with ESC for different time periods (7, 14, 21 days). At the starting time, cell monolayer immunophenotype was similar to isolated primary NPI. To monitor progress of the incubated cell differentiation, we have examined either markers of endocrine cell phenotype differentiation or c-kit, a hematopoietic stem cell marker, which has recently been involved with growth and differentiation of *β*-cell subpopulations in humans as well as rodents. In fact, tyrosine-kinase proteins are involved in growth and differentiation of different cell types. Among such proteins, a typical hematopoietic stem cell marker [12–14], whose ligand is stem cell factor (SCF) has progressively fueled hope on its possible role in developmental biology of islet cells [14]. Furthermore, c-kit in the form of RNA transcripts has been found in a *β*-cell subpopulation, suggesting that mature *β*-cells may derive from c-kit positive cells, within cell regeneration and neogenesis pathways [15, 16]. Even if c-kit has already been shown in rodents and also in humans by Li et al. [17], our data are, to our knowledge, the first to be observed in pigs, a potential and promising xenogenic cell source for cell therapy of T1DM, because of the limited availability of cadaveric human donor pancreata.

In summary, the present paper describes the possible mechanisms by which SC may induce rapid and significant maturation and differentiation of NPI cell monolayers into functionally competent *β*-cells.

## 2. Materials and Methods

### 2.1. Isolation of Neonatal Porcine Islets

NPIs were isolated according to previously established methods [3, 4] from Neonatal “Large White” pigs (birth time range: 1–3 days). Briefly, the piglets were anesthetized with 0.1 mg/Kg azaperon (Stresnil, 40 mg/mL, Janssen, Bruxelles, Belgium) and 15 mg/Kg ketamine (Imalgene, 100 mg/mL, Gellini Farmaceutici, Aprilia, Italy) coadministered intramuscularly. The piglets underwent total laparotomy, by midline incision, in order to carefully remove the pancreas. To prevent bacterial contamination, particular care was taken to avoid bowel nicking. Upon transportation to the laboratory in Eurocollins (SALF, Bergamo, Italy) on ice, the pancreas was cut into small pieces (1–3 mm^3^) and washed in Hank's balanced salt solution (HBSS) (Sigma Chemical Co, St. Louis, Mo, USA) according to previously reported methods. The tissue was finely minced and thereafter shaken in a collagenase solution (collagenase P, Roche, Milano, Italy) and subsequently washed twice in HBSS (Sigma Chemical Co) supplemented with 100 U/mL penicillin +0.1 mg/mL streptomycin (Sigma Chemical Co). Finally, the tissue was resuspended in HAM-F12 (EuroClone, Wetherby, UK) supplemented with 0.5% bovine serum albumin, fraction V (BSA) (Sigma Chemical Co), 50 *μ*M 3-isobutyl-1-methylxanthine (IBMX) (Sigma Chemical Co), 10 mM nicotinamide (Sigma Chemical Co), 2 mM L-glutamine (Sigma Chemical Co), and penicillin +0.1 mg/mL streptomycin (Sigma Chemical Co) and plated in 100 × 15 mm Petri dishes (Becton Dickinson Labware, Lincoln Park, NY, USA) (10 000 NPCC/plate).

### 2.2. Culture Maintenance of Neonatal Porcine Islet Monolayers

At day 4 of the isolation, NPI viability was assessed by staining the preparation with ethidium bromide (EB) (Sigma Chemical Co) and fluorescein-diacetate (FDA) (Sigma Chemical Co), as previously described [18]. NPI were than replated in Click's medium (Sigma Chemical Co) supplemented with 10% fetal bovine serum (EuroClone, Wetherby, UK), 0.5% bovine serum albumin, fraction V (BSA) (Sigma Chemical Co), 10 mM nicotinamide (Sigma Chemical Co), 2 mM L-glutamine (Sigma Chemical Co), and penicillin +0.1 mg/mL streptomycin (Sigma Chemical Co) using T25 tissue flasks for adherent cell growth (Greiner Bio-one, Frickenhausen, Germany), at a concentration of 30 NPI/flask. Upon adhesion to flask, NPI lost their normal three-dimensional architecture and formed cell monolayers.

### 2.3. Isolation of Sertoli Cells

SCs were isolated from neonatal prepubertal “Large-White” pigs, aging 7–10 days, according to the previously established methods, slightly modified in our laboratory [5, 19].

Briefly, after anesthesia, the testes were removed and finely minced prior to undergoing stepwise enzymatic digestion: first step with Collagenase P (Roche Diagnostics, S.p.A., Monza, Italy) in HBSS (Sigma Chemical Co) to dissociate the seminiferous tubules; second step with trypsin +DNAse I in HBSS. The tissue digest, resuspended in glycine to eliminate the residual Leydig and peritubular cells [20], was collected and culture-maintained in HAM F12 (EuroClone, Wetherby, UK) supplemented with 0.166 nM retinoic acid (Sigma Chemical Co) and 5 ml/500 mL of insulin-transforming selenium (ITS) medium (Becton Dickinson, NJ, USA), in 95% air/CO_2_ at 37°C. Upon 3 days of in vitro culture maintenance, SCs were incubated with 10 mM tris(hydroxymethyl)aminometane hydrochloride buffer (TRIS) (Sigma Chemical Co), as previously reported in the literature, in order to eliminate the residual germinal cells [21].

### 2.4. Encapsulation of Sertoli Cells within Alginate-Based Microcapsules

SC were encapsulated in alginate-based microcapsules according to our previously published methods [22–24]. Briefly, confluent SC monolayers (20 × 10^6^ cells/T75 flask) were scraped off the plate upon incubation with 0.05% trypsin/ethylenediaminetetraacetic acid (EDTA) (Gibco, Grandisland) (2 minutes), then washed, counted by hemocytometer, and tested for viability. SCs were suspended in 1 mL of 1.8% aqueous solution of in-house highly purified sodium alginate (Na-AG) (Stern Italia, Milano, Italy). The AG/SC suspension was continuously aspirated by a peristaltic pump, at a flow rate of 12–14 mL/min, and extruded through a mono-air-jet device (air flow rate: 5 l/min) under sterile conditions. The alginate suspension was continuously stirred to prevent cell clumping, which would possibly lead to inhomogeneous SC distribution within microcapsules. The formed microdroplets were collected on a BaCl_2_ bath (1.2%, w/v) which immediately turned them into gel microspheres, washed twice in saline, and employed upon 24 hours of in vitro culture maintenance. Before and after microencapsulation, SC viability was assessed by staining the preparations with ethidium bromide (EB) (Sigma Chemical Co) and fluorescein-diacetate (FDA) (Sigma Chemical Co), under fluorescence microscopy, as previously described [18].

### 2.5. Insulin Secretory Patterns of NPI Monolayers

The obtained NPI cell monolayers (originating from 30 NPI/T25 flask) were cocultured for 7, 14, 21 days with microencapsulated SC in Click's medium (Sigma Chemical Co) supplemented with 10% fetal bovine serum (EuroClone, Wetherby, UK), 0.5% bovine serum albumin, fraction V (BSA) (Sigma Chemical Co), 10 mM nicotinamide (Sigma Chemical Co), 2 mM L-glutamine (Sigma Chemical Co), and penicillin +0.1 mg/mL streptomycin (Sigma Chemical Co). The study was repeated in triplicate. Media collected from the flasks, during culture maintenance, were centrifuged at 300 g for 5 minutes. The cell supernatants were stored at −20°C before undergoing insulin assay by RIA (Myria, Milano, Italy). Glucose-stimulated insulin release (GSIR) was determined upon stepwise, 90 minutes, sequential exposure of the tissue to 50 mg/dL (2.66 mM) −300 mg/dL (16.7 mM) −50 mg/dL (2.66 mM) D-glucose at 37°C, in 95% air/CO_2_. The total obtained insulin was then normalized by the total cell number and/or insulin+ cell number. Total cell number was determined by staining cell nuclei with crystal violet, solubilizing the adsorbed dye into a solution of Triton X-100 and assessing optical density with a spectrophotometer [25]. Insulin+ cell number was determined by immuhocytochemistry (see Section 2.6). In addition, the ratio of insulin content to total cell protein content (*μ*U/mg protein) of NPI cell monolayers cultured alone or in the presence of ESC was determined. Briefly, whole cellular extracts, prepared for western-blotting analysis (see Section 2.7), were employed for insulin assay by RIA and for total protein determination according to Bradford method [26]. Data are reported as average of 3 independent experiments.

### 2.6. Immunocytochemistry

Control and ESC cocultured NPI cell monolayers were deposited on glass coverslips, washed with PBS, and fixed with 4% paraformaldehyde in Dulbecco's phosphate buffer (PBS) (EuroClone, Wetherby, UK). Cells were washed in PBS/1% BSA thrice, then incubated with 10% goat serum in PBS/1% BSA for 30 minutes to block nonspecific IgG binding. Upon dilution in PBS/1% BSA and transfer into glass coverslips, the specific primary antibodies, namely, 1 : 200 rabbit anti-mouse/rat pancreatic and duodenal homeobox gene 1 protein (PDX-1) (Chemicon International, Temecula, CA), 1 : 150 rabbit antiglucotransporter-2 (Glut-2) (Chemicon International, Temecula, Calif, USA), 1 : 200 guinea pig anti-human insulin (Linco, St. Louis, Mo, USA), 1 : 50 monoclonal mouse anti-human cytokeratin 7 (Ck7) (Dako Cytomation, Glostrup, Denmark), 1 : 50 rabbit anti-human glucokinase (GK) polyclonal antibodies (Santa Cruz Biotechnology Inc., Santa Cruz, Calif, USA), and mouse anti-porcine c-kit (kind gift by Dr. Dominguez, Madrid, Spain) were incubated overnight at 4°C in a humidified chamber. The coverslips were washed thrice with PBS/1%BSA for 5 minutes, then incubated with secondary antibodies (AlexaFluor 488 goat anti-rabbit, AlexaFluor 488 goat anti-mouse, working solution 1 : 500) (Molecular Probes, Carlsbad, Calif, USA), (Cy3 donkey anti-mouse, Cy3 donkey anti-rabbit working solution 1 : 500, Amersham International, Otelfingen, Switzerland), (rabbit anti-guineapig TRITC working solution 1 : 320, (Sigma Chemical Co). The coverslips were washed thrice with PBS/1%BSA, then mounted and examined under fluorescence microscopy (Nikon Eclipse TE2000-U) at 492 ± 15 nm for AlexaFluor 488 and 552 ± 15 nm for Cy3. The image processing and percentage of immunopositive cells (%) were determined, by using the software Nikon Eclipse EZ C1, version 2.30, by two independent investigators. For each experimental condition, 20 different images, containing at least 300 cells in total, were counted. In every image, all the cells were counted and subdivided into different groups if labeled for green fluorescence or red fluorescence, or both (yellow fluorescence). The experiment was repeated in triplicate.

### 2.7. Western-Blotting Analysis

Taking into consideration the important effects of ESC on viability and functional competence of NPI monolayers, the effects of ESC on NPI differentiation markers were investigated by immunohistochemistry.

Various differentiation markers were examined (see Table 1).

Whole cellular extracts were prepared as follows: cells obtained from NPI monolayers were resuspended in 100 *μ*L of 10 mM TRIS base (Sigma Chemical Co) at pH 7.4, 150 mM NaCl, 1 mM EDTA, 1 mM ethyleneglycol Bis(aminoethylether) Tetraacetic acid (EGTA), 1% v/v Triton X-100 (Sigma Chemical Co), 0.5% (v/v) Nonidet P-40 (Sigma Chemical Co), 1 M NaF (Sigma Chemical Co), 0.2 M NaO_3_V (Sigma Chemical Co), and 0.2 M phenylmethanesulfonylfluoride (Sigma Chemical Co). The mixture was then spun at 1000 g (Mikro 200, Hettich zentrifugen, Tuttlingen, Germany) for 10 minutes, the supernatant was collected and the total protein content determined by the Bradford method [26]. Small sample aliquots were stored at −20°C for Western blotting analysis.

Cell extracts were fractioned by 4–12% sodium dodecyl sulphate polyacrylamide gel electrophoresis (SDS-PAGE), 50 *μ*g protein/lane, blotted on nitrocellulose membrane (Biorad, Hercules, Calif, USA), and incubated overnight in buffer containing 10 mM TRIS, 0.5 M NaCl, 1% (v/v) Tween 20 (Sigma Chemical Co), 1 : 1000 anti-PDX-1 Ab (Chemicon International, Temecula, CA), 1 : 200 anti-GK Ab (Santa Cruz Biotechnology Inc., Santa Cruz, Calif, USA), 1 : 2500 anti-Glut-2 Ab (Chemicon International), 1 : 1000 anti-actin Ab (Sigma Chemical Co). The Ag-Ab complex was then detected by incubating the membrane for additional 60 minutes in buffer containing 1 : 5000 horse radish peroxidase-conjugated anti-rabbit IgG secondary Ab (Sigma Chemical Co). Specific bands were detected by ECL (enhanced chemiluminescence).

### 2.8. RT-PCR

Total RNA was isolated from cells obtained from NPI cell monolayers by Invisorb Spin-cell RNA mini-kit (Invitek GmbH, Berlin, Germany). RT was performed by Sprint Power Script PrePrimed SingleShots kit (Clontech, Palo Alto, Calif, USA). Oligos sequences used are listed as forward then reverse, 5′ to 3′: *β* *-actin *  5′-ATGGTGGGTATGGGTCAGAA-3′ and 5′-CTTCTCCATGTCGTCCCAGT-3′ amplify a product of 123 bp, *Nkx6.1 *  5′-AGGATCCATTTTGTTGGACA-3′ and 5′-CGCCAAGTATTTCGTTTGTT-3′  amplify a product of 111 bp, *PDX-1 *  5′-AGAGCCCGAGGAGAACAAG-3′ and 5′-GCGGCCTAGAGATGTATTTG-3′ amplify a product of 100 bp, *Glut-2 *  5′-CCGAGTTTTTCAGTCAAGGA-3′  and 5′-AGTCCGCAATGTACTGGAAG-3′ amplify a product of 109 bp, *GK *  5′-TAGAGCAGATCCTGGCAGAG-3′ and 5′-GTAGGTGGGCAGCATCTTC-3′ amplify a product of 99 bp, *NeuroD/Beta2 *  5′-CCTGTGCACCCCTACTCTTA-3′and 5′-TGCAGGATAGTGCATGGTAA-3′ amplify a product of 272 bp, *Insulin *  5′-CTTCTTCTACACGCCCAAGG-3′  and 5′-CGGCCTAGTTGCAGTAGTTC-3′ amplify a product of 190 bp, *c-kit *  5′-ACAAATCCATGCCCACACCCT-3′  and 5′-TTAAGCCGTATGCAGTGGCCTC-3′  amplify a product of 293 bp.

RT-PCR analyses were performed in the Mx3000P Instruments (Stratagene, La Jolla, Calif, USA) in a total volume of 20 *μ*L reaction mixture, following the manufacturer's recommendations, using the Brilliant SYBR Green QPCR Master Mix 2x (Stratagene) and 10 *μ*M of each primer using the dissociation protocol. Negative controls contained water instead of first-strand cDNA. Each sample was normalized on the basis of its housekeeping gene (*β*-actin). The relative gene expression levels were normalized to a calibrator that was selected as to be the control sample (untreated). Final results, expressed as relative expression, were calculated by MxPro software (Stratagene).

### 2.9. Statistical Analysis

All data were expressed as mean ± SD of three independent experiments. Statistical analysis was conducted by ANOVA. *P*- values < .05 were considered significant.

## 3. Results

### 3.1. Effects of Encapsulated Sertoli Cells on NPI Cell Monolayers: Morphology and Insulin Secretory Patterns

**Figure 1:**
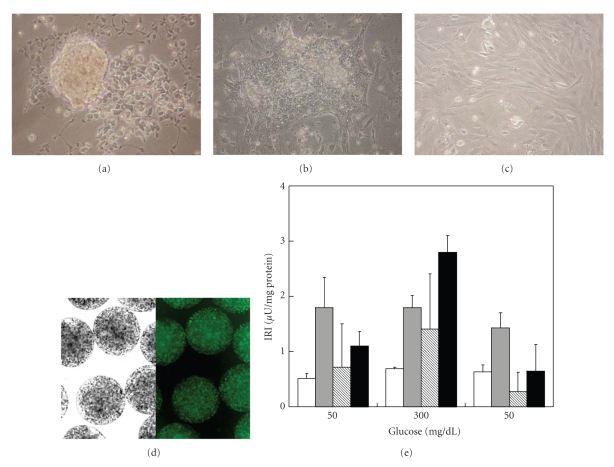
(a)–(c) Photomicrographs of NPI after culture for 1 (a), 6 (b), and 10 (c) days on T25 tissue flasks for adherent cell growth. (d) Light field (left) and fluorescence (right) photomicrographs of Ba-AG microcapsules containing SC. Fluorescence micrographs were obtained after staining with EB+FDA to assess SC viability. (e) Insulin secretory patterns of control NPI cell monolayers alone after 14 days (open bars) or NPI cell monolayers, cocultivated with microencapsulated SC for 7 (gray bars), 14 (hatched bars), and 21 days (filled bars) of culture. During static incubation, the cells were treated with the indicated concentrations of glucose. Data represent the average of 3 independent experiments; each insulin determination was performed in triplicate ±SD.

As reported by Figures 1(a) and 1(d), NPI adhesion process started at day 1 throughout complete adhesion, in 7–10 days. No appreciable morphological differences were detectable between NPI monolayers cultivated alone (data not shown) and those cocultivated with ESC throughout day 21. No significant differences have been observed between NPI cell monolayer cocultivated and NPI cell monolayer alone (data not shown). As shown by Figure 1(d), ESC cocultivated with NPI monolayers exhibited excellent morphology and cell organization, with the cell viability testing higher than 80% at day 21.

**Figure 2:**
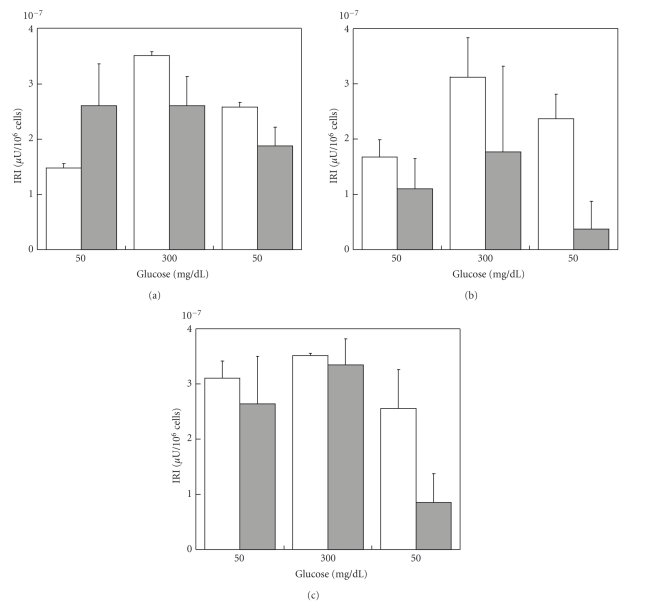
Ratio insulin/total cell number of control NPI cell monolayers alone (open bars), or NPI cell monolayers cocultivated with microencapsulated SC (gray bars) for 7 (a), 14 (b), and 21 days (c). Data are shown as means ± SD from 3 samples.

**Figure 3:**
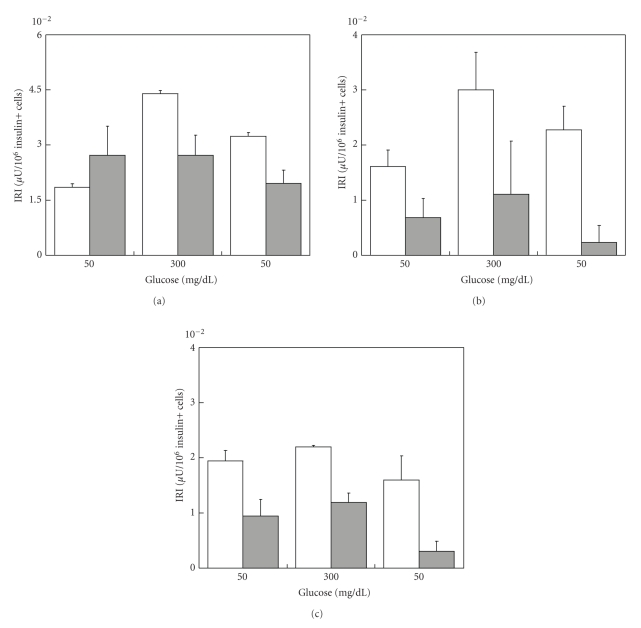
Ratio insulin/insulin+ cell number of control NPI cell monolayers alone (open bars), or NPI cell monolayers cocultivated with microencapsulated SC (gray bars) for 7 (a), 14 (b), and 21 days (c). Data are shown as means ± SD from 3 samples.

ESC strongly influenced insulin secretory patterns of NPI (Figure 1(e)). In fact, for the entire duration of the culture period (up to 21 days) the NPI cell monolayers cultivated alone, showed only limited insulin secretion (<0.7 *μ*U/mg protein), while control NPI showed a clear lack of insulin response to glucose (at 50-300-50 mg/dL). By sharp contrast, NPI cocultivated with ESC showed progressively better GSIR. At day 21, the cocultivated NPIs were fully responsive to glucose, and most importantly, were highly responsive to variations of the glucose levels (*P* < .05). As reported by Figures 2 and 3, the ratio insulin/total cell number and/or insulin+ cell number decreased in NPI cell monolayers cocultivated with ESC up to 21 days as compared to NPI cell monolayers alone, possibly because the increased insulin release reflects the presence of more insulin expressing cells in the treated cell monolayers. 

The ratio insulin content/mg total protein, being insulin content indicative of the differentiation/maturation of the NPI cell monolayers and total cell protein content indicative of the quantity of tissue, showed significant progressive increase of the endogenous insulin synthesis in the NPI cell monolayers cocultured with ESC up to 21 days as compared to NPI cell monolayers cultured alone (Figure 8(c)).

### 3.2. Effects of Encapsulated Sertoli Cells on NPI Cell Monolayers: Evaluation of Differentiation Markers by Immunohistochemistry

**Figure 4:**
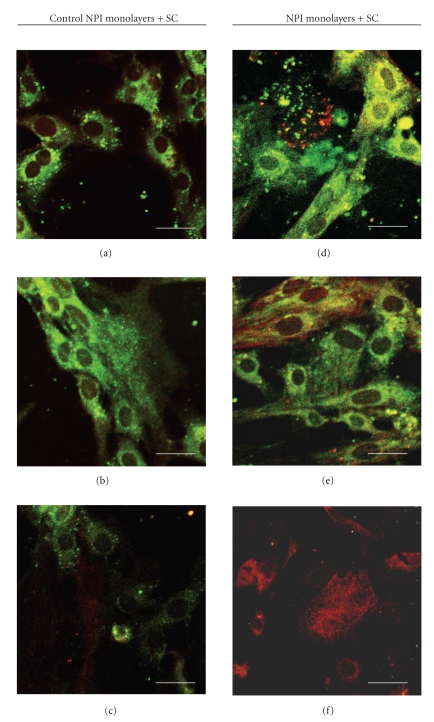
Double fluorescence immunolabeling (green signal: anti-CK-7 Mo-Ab; red signal anti-insulin polyclonal Ab) under confocal laser microscopy of NPI cell monolayers cultivated for 7 (a), (d), 14 (b), (e), and 21 (c), (f) days, alone (a)–(c) or with SC (d)–(f). Bar =10 *μ*m.

**Figure 5:**
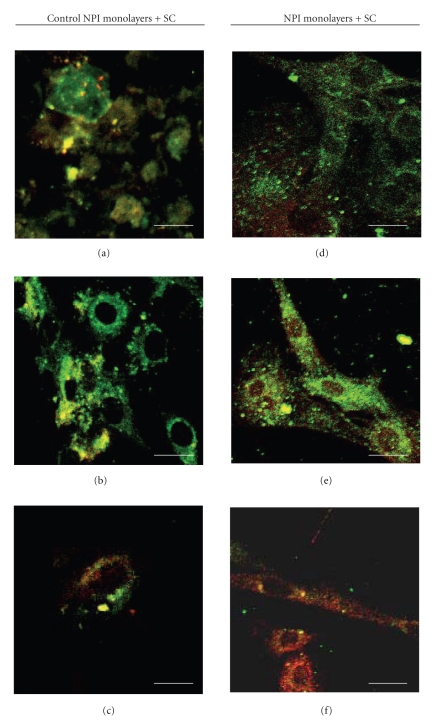
Double fluorescence immunolabeling (green signal: anti-PDX-1 Ab; red signal anti-insulin Ab) under confocal laser microscopy of NPI cell monolayers cultivated for 7 (a), (d), 14 (b), (e), and 21 (c), (f) days, alone (a)–(c) or with SC (d)–(f). Bar =10 *μ*m.

**Figure 6:**
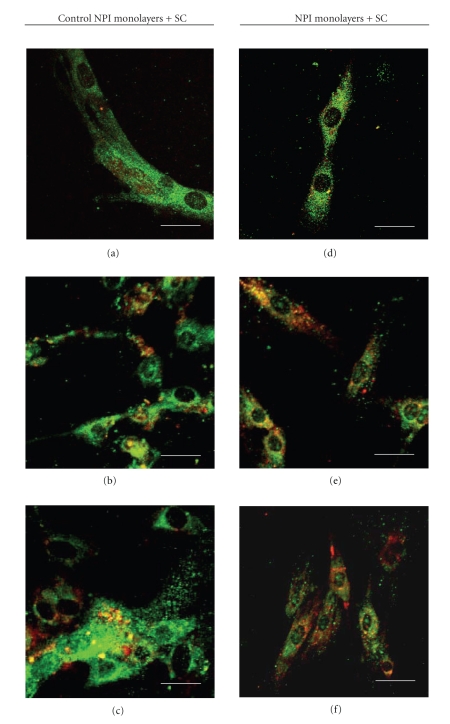
Double fluorescence immunolabeling (green signal: anti-PDX-1 Ab; red signal anti-c-kit Ab) under confocal laser microscopy of NPI cell monolayers cultivated for 7 (a), (d), 14 (b), (e), and 21 (c), (f) days, alone (a)–(c) or with SC (d)–(f). Bar =10 *μ*m.

**Figure 7:**
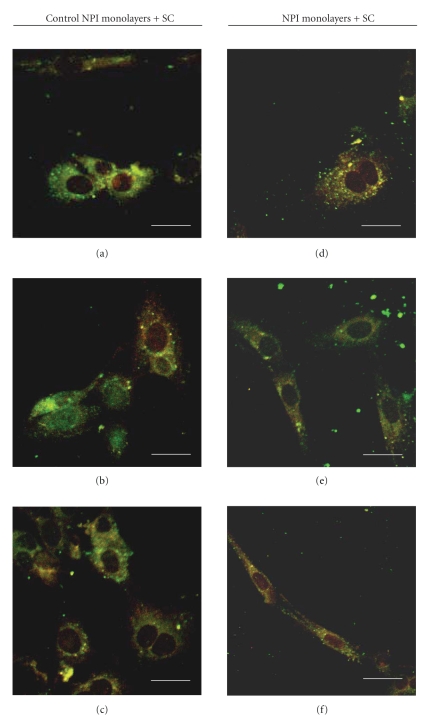
Double fluorescence immunolabeling (green signal: anti-c-kit Ab; red signal anti-insulin Ab) under confocal laser microscopy of NPI cell monolayers cultivated for 7 (a), (d), 14 (b), (e), and 21 (c), (f) days, alone (a)–(c) or with SC (d)–(f). Bar =10 *μ*m.

Different samples of control (untreated) NPI cell monolayers and ESC cocultured NPI underwent double immunostaining with different couples of primary antibodies, namely: Ck7/Insulin (Figure 4), PDX-1/Insulin (Figure 5), PDX-1/c-kit (Figure 6), Insulin+/c-kit (Figure 7). 

Immunophenotype of the primary isolated NPI was similar to the NPI cell monolayer patterns at 7 days (data not shown). 

Fluorescence micrographs reported in Table 2 showed that (a) the percentage of cells positive for both Ck7 and Insulin, an immature cell phenotype, which would likely suggest ductal origin of the *β*-cells, (Figure 4) declined progressively in the NPI cell monolayers cocultured with ESC, as compared to untreated NPI cell monolayers however, difference was statistically significant only for the 21 day-treated NPI (*P* < .050); (b) the percentage of NPI double positive for PDX-1/insulin, a mature *β*-cell phenotype (Figure 5), and insulin/c-kit, a cell phenotype associated with functionally immature *β*-cell subpopulations, significantly raised in the treated NPI populations both at days 14 and 21 (*P* < .050) (Figure 6), finally (c) the percentage of cells positive for PDX-1/c-kit (Figure 7) raised for the cocultured NPI, but only at day 21. The difference between untreated NPI and ESC-treated NPI was statistically significant (*P* < .050).

### 3.3. Effects of Encapsulated Sertoli Cells on NPI Cell Monolayers: Evaluation of Differentiation Markers by Western Blot Analysis

**Figure 8:**
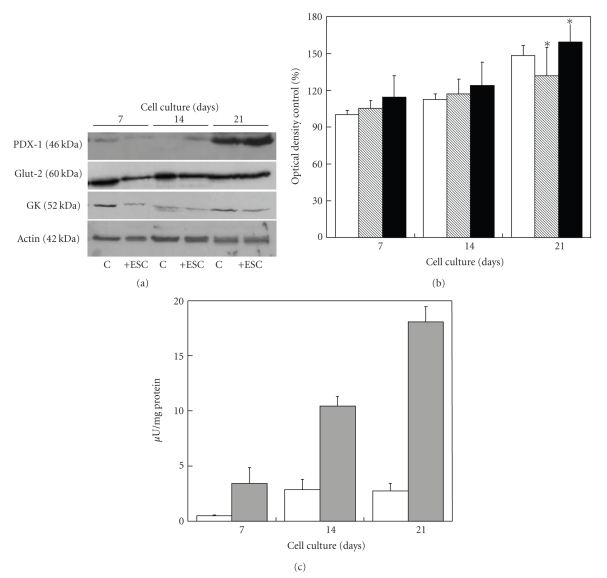
(a) Western blot analysis of the indicated proteins from control NPI cell monolayers (C) or NPI cell monolayers cocultured with ESC (+ESC) for the indicated time periods. (b) The relative intensities of PDX-1 (open bars), Glut-2 (hatched bars), and GK (filled bars) levels, as determined by band densitometric analysis. The ratio of the band intensities was expressed as a percentage with respect to untreated control NPI cell monolayers. Data are shown as means ± SD from 3 samples. *Significant difference between two groups at each time point (*P* < .05). (c) Ratio insulin content/mg total protein of control NPI cell monolayers alone (open bars), or NPI cell monolayers, cocultivated with microencapsulated SC (gray bars), for 7, 14, and 21 days of culture. Data are shown as means ± SD from 3 samples.

To confirm the data obtained by immunocytochemistry, a new set of experiments was planned by the complementary western blotting technique. The results of these experiments are reported in Figure 8 where both photographs of the nitrocellulose membranes and the barplots of the densitometric analysis are exhibited. The Western blots confirmed statistically significant differences between control NPI and NPI cocultered in the presence of ESC. In particular, cocultured NPI showed a statistically significant increase of the expression of PDX-1, Glucokinase (GK), and GLUT-2 as compared to controls (*P* < .050).

### 3.4. Effects of Encapsulated Sertoli Cells on NPI Cell Monolayers: Assessment of Differentiation Markers by Real Time PCR (qPCR)

**Figure 9:**
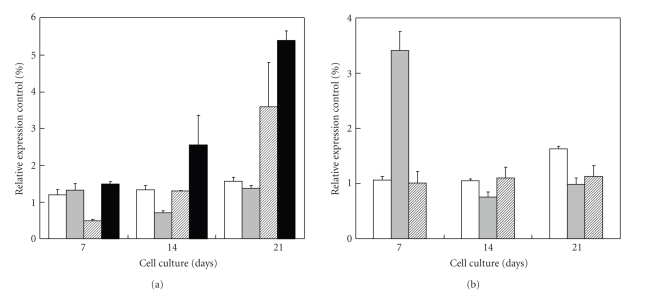
qPCR analysis of the indicated genes from NPI cell monolayers cocultivated in the presence of ESC for the indicated time periods. (a) PDX-1 (open bars), NeuroD (gray bars), Nkx6.1 (hatched bars), and c-kit (filled bars). (b) Insulin (open bars), Glut-2 (hatched bars), and GK (filled bars). The reported results are expressed as a percentage out of untreated control NPI cell monolayers. Data are shown as means ± SD from 3 samples.

qPCR analysis, reported by Figure 9, showed statistically significant differences in the expression of a number of genes between treated and untreated NPI. At day 21, the cocultured NPI showed remarkable increase in the expression of PDX-1, NKx6.1, Insulin, and c-kit genes (*P* < .050). At days 7 and 21, the cocultured NPI showed statistically significant differences for Neuro D and Glut-2 as compared to controls (*P* < .050), meanwhile only at day 7 statistically significant differences for Glut-2 were observed (*P* < .050). For Gk, no statistical significant differences were shown (data not shown).

## 4. Discussion

The restricted availability of cadaveric human donor pancreata in conjunction with poor results of the Immune Tolerance Network (ITN) multicentric clinical trial on human islet cell transplantation [3] has considerably downsized the impact of human islet transplantation on the possible cure of TIDM. Moreover, possible risks for malignancies in patients with diabetes treated with human insulin or insulin analogues have recently been reported [2]. For these reasons, new sources of insulin-producing cells are actively being sought [4, 5]. In this respect, we had previously shown that SCs are able to induce either mitogenic activity of adult rat islet-beta cells [27] or rapid and significant maturation and differentiation of freshly isolated NPI into functionally competent *β*-cells [5].

Freshly isolated NPI cell populations are typically comprised of a minority of *β*-cells, a majority of CK7+ cells, while the remainder cell population coexpresses both insulin and epithelial cell markers. In fact, as reported by Trivedi et al. [7] and Korbutt et al. [4], NPI may take 4–10 or more weeks to reach as sufficient, differentiated *β*-cell mass as to enable reversal of hyperglycemia after TX in diabetic rodents. After in vitro exposure to SC, double fluorescence immunolabeling clearly showed that epithelial cells, stained with anti-CK7 MoAb, initially representing over 60% of the total cell population, turned into insulin-positive cells (74% as compared with 6% control NPI). Such an acceleration of the islet cell maturation process, induced by 9-day SC coculture, and substantiated by functional insulin data, was observed [5]. This process is limited by the NPI mass loss during the experimental procedure indeed. Hence, starting from NPI, we were able to generate long-lasting NPI cell monolayers, with no use of extracellular matrices or cell engineering approaches [11]. In this respect, NPI cell monolayers could serve as an experimental tool to assess the effects of several growth factors on *β*-cell molecular pathways, possibly allowing to examine the islet cell lineage commitments and to expand the starting cell material [11].

The achieved results showed, for the first time, that NPI cell monolayers were associated with c-kit+ cells in accordance to previous reports in mice and rats [15, 16, 28]. Confocal microscopy examination showed c-kit+/PDX-1+ and c-kit+/insulin+ cells that might represent progenitors, and possibily *β* cell precursors. In fact, during pancreatic development, differentiated cells derive from the PDX-1+ ductal precursor cells: consequently, colocalization of c-kit/PDX-1 could coincide with endocrine *β* cell precursors here, at 21 days of coculture, when c-kit and PDX-1 transcription factors are upregulated in a pancreatic neogenesis model. NeuroD/ *β*2 and NKx6.1 promoter mRNA showed that during cell coculture, the cells shifted toward a more mature phenotype. Glut-2 mRNA tended to decline at 14 and 21 days of coculture, possibly indicating protein deregulation. Unlike some authors [29] and according to others [30], we have observed, in our cell monolayers, c-kit and insulin colocalization by factor 3-4 at 21 days of culture. WB densitometric analysis has revealed an increase of phosphorylated PDX-1 in the treated (statistically significative at 21 days) as compared to the control monolayers. It is known that PDX-1 regulates the insulin gene expression, sinergistically with other factors belonging to the helix-loop-helic basic protein family. The resulting heterodimeric complex binds to the E2 element of the insulin promoter [11]. This data has been confirmed by qPCR indicating that at 21 days there is an insulin mRNA active transcription in conjunction with the highest insulin content/mg total protein ratio. However, not all insulin positive cells were also marked positively for c-kit [16] thereby suggesting that these cells could embody a subset of endocrine precursor cells.

One of the possible mechanisms of action could be related to SCF secretion. SCs produce SCF or c-kit ligand that binds to and activates the transmembrane tyrosine kinase receptor c-kit. SCF/c-kit interaction plays a very important role in the development, function, and survival of rodent islets of Langerhans [14, 15]. SCF has been shown to promote an increase of insulin output in fetal rat islets [15]. Interestingly, coculture of our cell monolayers with microencapsulated SC induced an increase in PDX-1+/insulin+ and c-kit+/insulin+ cell percentage, according to previous observations by the use of SCF [31] in either fetal human [17] or rat [28] islet experimental settings. Likewise, an increase in PDX-1 and c-kit mRNA also was observed. 

We found a significant increase of endogenous insulin output, under glucose stimulation, from the NPI cell monolayers that were cocultured with SC, as compared to NPI cell monolayers alone. In fact, static incubation clearly documented that ESC-treated cell monolayers responded physiologically to glucose changes within 90 minutes, differently from controls that did not undergo comparable maturation patterns. The decreasing ratio insulin/total cell number and/or insulin+ cell number in NPI cell monolayers cocultivated with ESC, up to 21 days, as compared to NPI cell monolayers alone, probably reflects the increase of insulin expressing cells in the treated monolayers. Moreover, the ratio insulin content/mg total protein progressively increased for the entire culture time period (up to 21 days) likely indicating that ECS seem to promote maturation. ESC-derived SCF could induce the differentiation of islet cell precursors by different mechanisms, including phosphatidylinositol-3-kinase (PI3K9), the Janus family of protein tyrosine kinases, the Src family members, and the Ras-Raf-mitogen-activated protein (MAP) kinase. These pathways mediate several cellular processes, including increased gene transcription, proliferation, differentiation, survival, and metabolic homeostasis [32, 33].

In conclusion, our data seems to support the idea that microencapsulated SC may accelerate the differentiation of monolayered porcine cell cultures in the short term. This effect could be explained by the increase of SC-induced PDX-1+/insulin+ and c-kit+/insulin+ cell mass. Potential consequences of these observations, with respect to differentiation of mature porcine *β*- cells as a possibile xenogeneic cell source in diabetes, are implicit.

## Figures and Tables

**Table 1:** Cell type markers analyzed by immunocytochemistry on NPI cell monolayers cultured alone or with ESC.

Name	Abbreviation	Cell localization	Cell type marker
Stem cell factor receptor	c-kit/SCFR	transmembrane	hematopoietic stem cell marker; growth and differentiation of *β*-cell sub-populations marker
Cytokeratin 7	Ck7	cytoplasmatic	ductal cell marker
Insulin	Ins	cytoplasmatic	*β*-cell marker
Pancreatic and duodenal homeobox gene 1 protein	PDX-1	Nuclear and cytoplasmatic	*β*-cell marker

**Table 2:** Immunohistochemical analysis of differentiation markers of NPI monolayers cultured alone or in the presence of ESC.

Cells (days)	Ck7+/Insulin+ (%)	PDX-1+/Insulin+ (%)	Insulin+/c-kit+ (%)	PDX-1/c-kit+ (%)
C (7)	58.5 ± 1.4	14.8 ± 3.9	10.0 ± 1.7	13.1 ± 2.3
+ESC (7)	55.3 ± 5.5	14.3 ± 3.0	9.7 ± 4.0	14.3 ± 9.7
C (14)	50.0 ± 3.8	20.7 ± 3.1	14.0 ± 3.7	25.7 ± 10.0
+ESC (14)	40.2 ± 2.4	33.3 ± 5.1*	26.0 ± 0.9*	31.1 ± 13.5
C (21)	45.6 ± 4.7	29.5 ± 4.9	15.8 ± 2.9	45.1 ± 11.0
+ESC (21)	32.9 ± 4.1*	74.8 ± 4.9*	38.3 ± 5.2*	71.8 ± 10.8*

C: Control untreated NPI monolayers.

+ESC: NPI monolayers cocultured in the presence of encapsulated Sertoli cells.

Data represent the percentage of positive cells and are given as means ± SD (*n* = 3).

*P < .050.
